# The polymorphism analysis for CD36 among platelet donors

**DOI:** 10.1038/s41598-024-58491-z

**Published:** 2024-04-12

**Authors:** Qilu Lyu, Yuwei Lin, Yiming Pan, Xiaoyu Guan, Xin Ji, Mozhen Peng, Qian Li, Zhijang Wang, Zhihui Zhang, Zhen Luo, Pincan Su, Jue Wang

**Affiliations:** 1 Clinical Transfusion Research Center, Institute of Blood Transfusion, Chinese Academy of Medical Sciences and Peking Union Medical College, Chengdu, 610052 Sichuan China; 2 Key Laboratory of Transfusion Adverse Reactions, CAMS, Chengdu, 610052 Sichuan China; 3 School of Public Health, Anhui Medical University, Hefei, Anhui China; 4 Department of Blood Transfusion, The Third People’s Hospital of Chengdu, Affiliated Hospital of Southwest Jiaotong University, Chengdu, Sichuan China; 5 Yunnan Kunming Blood Center, Kunming, 650000 Yunnan China; 6 Department of Transfusion Medicine, Affiliated Hospital of Yunnan University, Kunming, 650000 Yunnan China

**Keywords:** Platelet donors, Polymorphism, CD36, sCD36, Immunology, Molecular biology, Molecular medicine

## Abstract

CD36 may defect on platelets and/or monocytes in healthy individuals, which was defined as CD36 deficiency. However, we did not know the correlation between the molecular and protein levels completely. Here, we aim to determine the polymorphisms of the *CD36* gene, RNA level, and CD36 on platelets and in plasma. The individuals were sequenced by Sanger sequencing. Bioinformational analysis was used by the HotMuSiC, CUPSAT, SAAFEC-SEQ, and FoldX. RNA analysis and CD36 protein detection were performed by qPCR, flow cytometry, and ELISA. In this study, we found c.1228_1239*del*ATTGTGCCTATT (allele frequency = 0.0072) with the highest frequency among our cohort, and one mutation (c.1329_1354*dup*GATAGAAATGATCTTACTCAGTGTTG) was not present in the dbSNP database. 5 mutations located in the extracellular domain sequencing region with confirmation in deficient individuals, of which c.284T>C, c.512A>G, c.572C>T, and c.869T>C were found to have a deleterious impact on CD36 protein stability. Furthermore, the MFI of CD36 expression on platelets in the mutation-carry, deleterious-effect, and deficiency group was significantly lower than the no-mutation group (*P* < 0.0500). In addition, sCD36 levels in type II individuals were significantly lower compared with positive controls (*P* = 0.0060). Nevertheless, we found the presence of sCD36 in a type I individual. RNA analysis showed CD36 RNA levels in platelets of type II individuals were significantly lower than the positive individuals (*P* = 0.0065). However, no significant difference was observed in monocytes (*P* = 0.7500). We identified the most prevalent mutation (c.1228_1239*del*ATTGTGCCTATT) among Kunming donors. Besides, our results suggested RNA level alterations could potentially underlie type II deficiency. Furthermore, sCD36 may hold promise for assessing immune reaction risk in CD36-deficient individuals, but more studies should be conducted to validate this hypothesis.

## Introduction

CD36, also known as glycoprotein IV (GPIV), is widely expressed on the surfaces of various cell types, including platelets, macrophages/monocytes, and adipocytes^1^. As a member of the class B scavenger receptor group, CD36 serves diverse physiological functions, encompassing lipid metabolism regulation^2^, involvement in thrombus formation on collagen^3^, and malaria-induced immune responses^4^. CD36 deficiency could occur in healthy individuals and has two subgroups: type I (defect on both platelets and monocytes) and type II (only defect on platelets)^5,6^. Individuals with CD36 deficiency, particularly type I, have an elevated risk of developing CD36 antibodies upon exposure to positive antigens through pregnancy, transfusion, or transplantation^7–10^. CD36 antibodies have a significant role in immune-mediated disorders, such as platelet transfusion refractoriness and fetal/neonatal alloimmune thrombocytopenia (FNAIT)^9,11^. Notably, CD36 antibodies are the primary pathogenic factor for FNAIT in China^12^. Intriguingly, recent literature demonstrates that CD36 conforms to the criteria for a new blood group, and anti-CD36 can also cause fetal anemia^13^, drawing more attention to focus on CD36.

The prevalence of CD36 deficiency varies significantly across regions, countries, and ethnic groups. Africans exhibit a higher incidence at 7.70%^14^, while Asians range from 1.68 to 8.20%^15,16^, and Caucasians have a lower prevalence at less than 0.40%^17^. Interestingly, there are notable regional and interethnic variations in CD36 deficiency within China. Southerners and ethnic minorities, such as the Man and Zhuang, have a frequent incidence of CD36 deficiency^18,19^. A previous study has demonstrated that 329–332 *del*AC is one of the most prevalent mutations in the Chinese population for CD36 deficient individuals, and the mutation is also frequently found in heterozygous form among CD36-positive individuals^17^.

CD36 is not only expressed on cells but also present in plasma, where it is referred to as soluble CD36 (sCD36)^20^. The sCD36 has displayed its potential as a plasma biomarker and has been linked to conditions such as atherosclerosis, metabolic syndrome, and malaria-infected erythrocytes. Alkhatatbeh et al.^21,22^ found that sCD36 is non-cleaved, non-soluble, and entirely associated with microparticles released from platelets and endothelial cells. More recently, a study by Phuangtham^23^ identified a significant correlation between CD36 on platelets and sCD36 in plasma, although the sample size was limited (N = 14). Intriguingly, this study did not find a substantial disparity in sCD36 levels between individuals with type II deficiency and those with a positive CD36 status.

In this study, our objective is to explore CD36 genetic polymorphism and assess genetic variations from DNA to protein levels (CD36 on platelets and in plasma). Additionally, we compare RNA level analysis between CD36-positive and CD36-negative individuals, combining to our previous study which reported seven cases of CD36 deficiency^16^.

## Materials and methods

### Sample preparation

Platelet donors were recruited from the Kunming Blood Center, following the inclusion criteria and specimen collection strategy outlined in our previous study^16^. A total of 418 platelet samples and 122 plasma samples were included in this study. All donors provided informed consent to participate in this study. The isolation of cells, DNA and RNA extraction, and cDNA preparation were carried out in accordance with established protocols as described in previous study.

### Flow cytometry analysis

The flow cytometry method was described in us previous study^16^. Briefly, CD36 expression on the cells was determined using flow cytometry (FC500, Beckman Coulter). Platelets were obtained from platelet-rich plasma and incubated with phycoerythrin (PE)-labeled anti-CD41 antibodies (Biolegend, San Diego, CA, USA). Monocyte detection utilized 7-amino-actinomycin D (7-AAD, BD Pharmingen) and PE-conjugated anti-CD14 antibodies (M5E2, BD Pharmingen). CD36 expression was determined using fluorescein isothiocyanate (FITC)-conjugated anti-CD36 antibodies (CB38; BD Pharmingen, San Diego, CA, USA).

### DNA analysis

The *CD36* gene comprises 15 exons, but only exons 3 to 14 are involved in protein coding. In our research, we examined mutations located within these protein-coding exons in all CD36-positive individuals. The primers, reaction systems, and polymerase chain reaction (PCR) conditions were detailed in the previous publication^16^. The obtained results were analyzed using Chromas 2.5 and aligned with the reference sequence (Accession Number: NG_008192).

### Prediction of the mutation causing amino acid substitution

To gain deeper insights into the impact of mutations involved in coding the extracellular domain, especially those not yet confirmed in deficiency cases, we employed HotMuSiC^24^, CUPSAT^25^, SAAFEC-SEQ^26^ and FoldX^27^ to assess the protein stability resulting from amino acid substitutions in individuals with missense mutations. Catalogizing the mutations which were predicted as destabilizing by three of these four tools, into the Predicted Group (PreGroup). Meanwhile, to validate the reliability of the predication tools, we also used the tools to predict the protein stability caused by 4 mutations (c.268C>T, c.410T>C, c.1156C>T and c.1163A>T), which has been known that could lead to CD36 deficiency.

### RNA level analysis

We analyized RNA using qPCR (Bio-Rad CFX96, Berkeley, CA, USA) in CD36-positive individuals who had CD36 deficiency-related mutations and CD36-negative individuals previously identified. According to our prior study, exon 2–4 could be skipped, therefore, we designed detection primers located at exon 5 and its junction with exon 6 (forward primer: TGGTGCCATCTTCGAACCTT; reverse primer: GGATGCAGCTGCCACAG). We selected β-actin as the reference primer (forward primer: TGGCACCCAGCACAATGAA; reverse primer: CTAAGTCATAGTCCGCCTAGAAGCA). The qPCR analysis utilized the FastStart Universal SYBR Green Master (ROX) kit (Roche, Basel, CH). Each detection was performed twice, and the relative CD36 RNA levels were calculated using the ∆∆Ct method.

### Detection of sCD36 in plasma

Plasma collection by centrifugation method with 3000*g* centrifugal force for 15 min, followed by removing the sediment and remaining the supernat. The obtained plasma was stored at – 80 °C. sCD36 was detected by Human platelet membrane glycoprotein IV, ELISA Kit (CUSABIO, Wuhan, CN), repeated twice for each sample.

### Statistical analysis

Continuous values, including the mean fluorescence intensity (MFI) of CD36 on platelets, the concentration of sCD36 in plasma, and the comparative expression of CD36 RNA levels, were presented as mean ± standard deviation (M ± SD). Statistical analysis was conducted using GraphPad Prism 9 (GraphPad Software Inc., San Diego, CA, USA). Differences between two groups were assessed using an independent t-test, with statistical significance defined as a P value less than 0.0500, and multiple groups comparison using One-way Anova. Correlation analysis between CD36 on platelets and sCD36 in plasma was performed using the Simple Linear Regression method, and the results were recorded, including P values and the coefficient of determination (r-value).

### Ethics approval and consent to participate

The study was approved by the ethics committee of the Institute of Blood Transfusion, Chinese Academy of Medical Sciences, and all methods were performed in accordance with the relevant guidelines and regulations.

## Results

### Polymorphisms analysis for *CD36* protein-coding region

**Figure 1:**
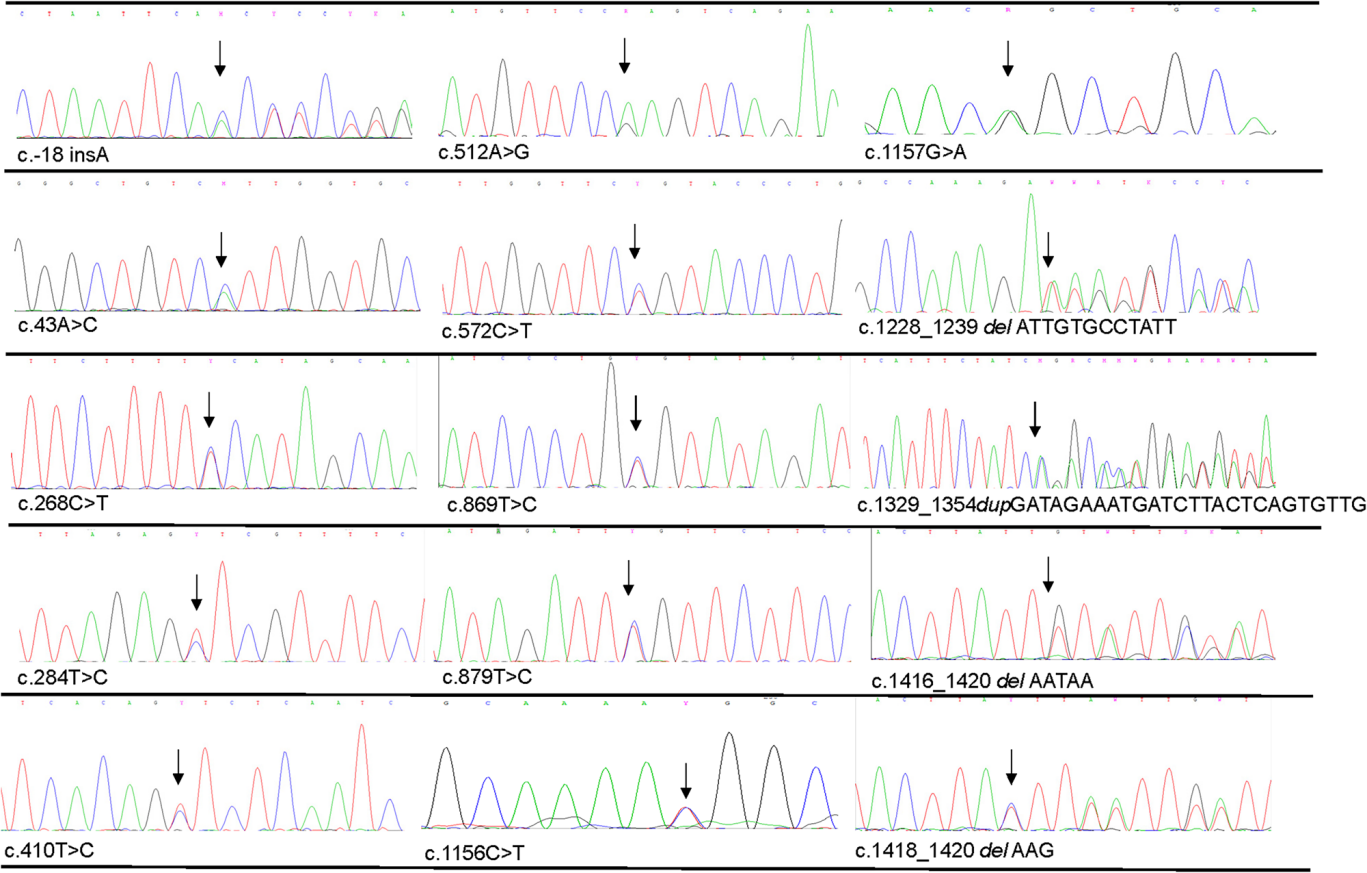
Sequencing chromatograms for the positive individuals harboring mutations. The position of the substitution/deletions/insertion is pointed by arrows. All the mutations are heterozygotes.

In our study, we included 418 platelet donors, among whom 7 individuals exhibited CD36 deficiency^16^. Exon 3–14 were investigated by Sanger sequencing among the CD36-positive individuals. The results showed that 15 heterozygous mutations were detected among 26 out of 411 CD36-positive platelet donors (Table 1; Fig. 1). 5 mutations (c.-18 insA, c.43A>C, c.1329_1354*dup*GATAGAAATGATCTTACTCAGTGTTG, c.1416_1420*del*AATAA, and c.1418_1420*del*AAG) were identified in the exons, which collectively cover the coding regions for the 5ʹ untranslated regions (UTR) and the cytoplasmic or transmembrane domains. Particularly, c.1329_1354*dup*GATAGAAATGATCTTACTCAGTGTTG, seems a new mutation and has not been described before. In addition, there were 9 single nucleotide polymorphisms (SNP) (c.268C>T, c.284T>C, c.410T>C, c.512A>G, c.572C>T, c.879T>C, c.869T>C, c.1156C>T, and c.1157G>A) and one deletion mutation (c.1228_1239*del*ATTGTGCCTATT) located at extracellular topological domain coding sequence region.

**Table 1:** Summary of polymorphisms of CD36 sequencing exons for platelet donors.

Number of individuals	Exon	Variant ID	Nucleotide change	Protein change	Mutation type	Protein location	CD36 deficency related report	Allele frequency
2	3	rs75112981	c.-18 *ins*A	–	Non coding transcript variant	5ʹ-UTR	–	0.0024
1	3	rs778170886	c.43A>C	I15L	Missense	Transmembrane	–	0.0012
2†	4	rs75326924	c.268C>T	P90S	Missense	Extracellular topological domain	Kashiwagi H, J Clin Invest, 1995^28^	0.0024
1	5	rs777437579	c.284T>C	V95A	Missense	Extracellular topological domain	–	0.0012
1†	5	rs572295823	c.329_330*del*AC	Protein alteration after mutation	Frameshift mutation	Extracellular topological domain	Kashiwagi H, Blood, 1994^29^	0.0012
1	5	rs2272350	c.410T>C	V137A	Missense	Extracellular topological domain	Imai M, Clin Chim Acta. 2002^5^	0.0012
1	6	rs1286758110	c.512A>G	Q171R	Missense	Extracellular topological domain	–	0.0012
1	6	rs143150225	c.572C>T	P191L	Missense	Extracellular topological domain	–	0.0012
1	10	rs770734754	c.869T>C	V290A	Missense	Extracellular topological domain	–	0.0012
5	10	rs188717259	c.879T>C	–	Nonsense	Extracellular topological domain	–	0.0060
2†	12	rs148910227	c.1156C>T	R386W	Missense	Extracellular topological domain	Okajima, Thromb Haemost, 2006^30^	0.0024
1	12	rs187500047	c.1157G>A	R386Q	Missense	Extracellular topological domain	–	0.0012
1†	12	rs201355711	c.1163A>T	Q388L	Missense	Extracellular topological domain	Xu X, Blood Transfusion, 2014^17^	0.0012
6†	13	rs550565800	c.1228_1239 *del*ATTGTGCCTATT	Deletion of Ile-Val-Pro-Ile	Frameshift mutation	Extracellular topological domain	Masuda Y, Thromb Res, 2015^15^	0.0072
1	14	not found	c.1329_1354*dup*GATAGAAATGATCTTACTCAGTGTTG	Protein alteration after mutation	Frameshift mutation	Transmembrane	–	0.0012
2	14	rs771061715	c.1416_1420*del*AATAA	Protein alteration after mutation	Frameshift mutation	Intracellular topological domain	–	0.0024
2	14	rs767398084	c.1418_1420*del*AAG	Protein extension	Terminator Codon variant	Intracellular topological domain	–	0.0024

^†^The mutation found in one case CD36 deficiency individual, and the individual is removed to the MFI analysis.

We calculated the allele frequencies for the identified mutations and observed that c.1228_1239 *delATTGTGCCTATT* has the most frequent incidence in our cohort (allele frequency = 0.0072) (Table 1). Among these mutations found in CD36-positive individuals, only c.268C>T, c.410T>C, c.1156C>T, and c.1228_1239*del*ATTGTGCCTATT have been confirmed in individuals with CD36 deficiency^5,15,28,30^. However, the effects of the rest of the mutations found in this study impacting on CD36 expression are uncertain, particularly those located in exon 4 to exon 13, which code for the extracellular domain and may impact the binding between the receptor and its ligand.

### Prediction of substitution mutation on exon 4 to exon 13 responsible for extracellular domain coding.

To conduct an in-depth analysis of mutations (c.284T>C, c.512A>G, c.572C>T, c.869T>C, and c.1157G>A) located within the extracellular domain of the *CD36* gene, we employed bioinformatic analysis through HotMuSiC, CUPSAT, SAAFEC-SEQ, and FoldX. We used the CD36-deficiency-related mutation to verify the efficiency of these 4 prediction tools (Table 2), and only HotMuSiC predicts c.1163A>T as a protein stabilizing mutation, suggesting the other 3 prediction models have a better analytical effect than HotMuSiC.

**Table 2:** Prediction for Mutation c.268C>T, c.410T>C and c.1156C>T.

Nucleotide change	Protein change	HotMusic	CUPSAT	SAAFEC-SEQ	FoldX	MFI
c.268C>T	P90S	Destabilizing	Destabilizing	Destabilizing	Destabilizing	10.4
c.410T>C	V137A	Destabilizing	Destabilizing	Destabilizing	Destabilizing	7.32
c.1156C>T	R386W	Destabilizing	Destabilizing	Destabilizing	Destabilizing	8.07
c.1163A>T	Q388L	Stabilizing	Destabilizing	Destabilizing	Destabilizing	–

“–” represents CD36 negative on platelet since c.1163A>T was detected in a CD36 type II deficiency individual^16^.

Given the classification criteria described in the method, we defined 4 of these mutations (c.284T>C, c.512A>G, c.572C>T, and c.869T>C) as a destabilizing mutation for protein, except for c.1157G>A with two tools display destabilizing mutation and two show stabilizing mutation (Table 3). Notably, a significant difference emerged between the MFI of these four individuals and the average MFI of all positive donors (*P* = 0.0400, 6.54 ± 3.84 vs 12.09 ± 5.47).

**Table 3:** Prediction for mutation c.284T>C, c.512A>G, c.572C>T, c.869T>C and c.1157G>A.

Nucleotide change	Protein change	HotMusic	CUPSAT predicted	SAAFEC-SEQ	FoldX	MFI
c.284T>C	V95A	Destabilizing	Destabilizing	Destabilizing	Destabilizing	4.55
c.512A>G	Q171R	Destabilizing	Stabilizing	Destabilizing	Destabilizing	10.91
c.572C>T	P191L	Destabilizing	Stabilizing	Destabilizing	Destabilizing	2.32
c.869T>C	V290A	Destabilizing	Destabilizing	Destabilizing	Destabilizing	8.36
c.1157G>A	R386Q	Stabilizing	Stabilizing	Destabilizing	Destabilizing	11.49

### MFI analysis of platelets among wild-type, mutation carrier, predicted mutation, and deficient individuals

**Figure 2:**
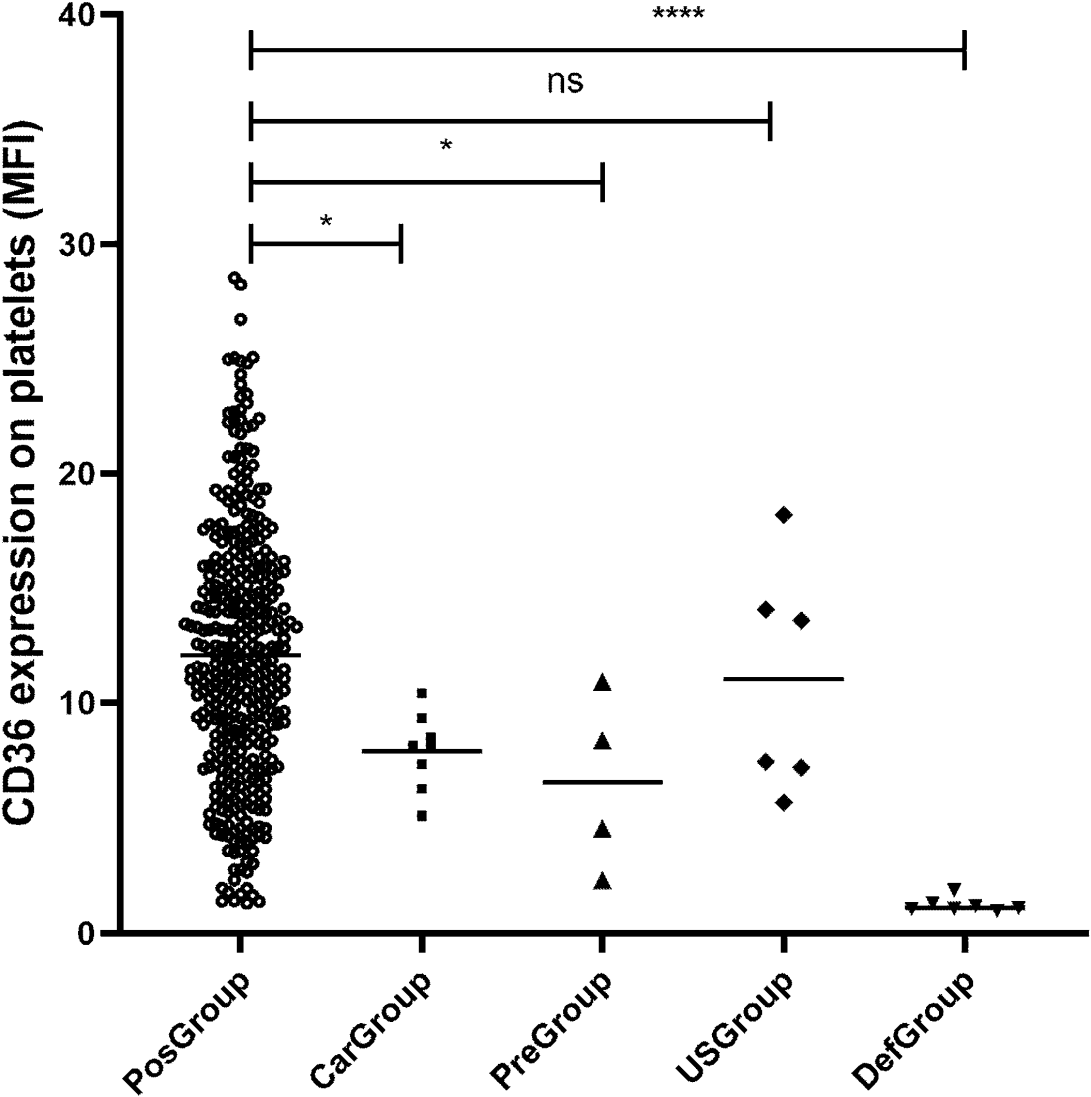
Analysis of CD36 expression on platelet. PosGroup indicates positive individuals without any mutation on Exon 3 to Exon 14 (N = 390); CarGroup represents that individuals carry CD36 deficiency-related mutations (N = 8); PreGroup indicates the deleterious mutations predicated by PoPMuSiC (N = 4); USGroup and DefGroup respectively represent the individuals containing mutation on transmembrane and endodomain sequencing area (N = 6), and CD36 deficiency individuals (N = 7). “*” and “ns” were used to show statistical significance. “*” and “****” respectively denote *P* < 0.0500 and *P* < 0.0001, while ns mean no significant difference.

Following a comprehensive DNA analysis and bioinformatic prediction, we categorized the 8 individuals harboring CD36 deficiency-associated mutations (c.268C>T, c.410T>C, c.1156C>T, and c.1228_1239*del*ATTGTGCCTATT) into the Carrier Group (CarGroup). In parallel, 4 individuals (c.284T>C, c.512A>G, c.572C>T, and c.869T>C) displaying a predicted deleterious stability, as determined by bioinformatics, were assigned to the Predicted Group (PreGroup). Additionally, we identified 6 individuals carrying mutations (c.43A>C, c.1329_1354*dup*GATAGAAATGATCTTACTCAGTGTTG, c.1416_1420*del*AATAA, and c.1418_1420*del*AAG) within the transmembrane and endodomain sequencing areas, and they were classified into the Unknown-Sense Group (USGroup). Notably, all subjects within these groups were CD36-positive individuals. In contrast, the Deficient Group (DefGroup) comprised 7 individuals^16^ with confirmed CD36 deficiency. Subsequently, comparing these groups with CD36-positive individuals who exhibited no mutations in Exon 3 to Exon 14 (PosGroup), respectively. The results are illustrated in Fig. 2. Specifically, the MFI of CD36 on platelets in CarGroup, PreGroup, and DefGroup was found to be significantly lower than that of PosGroup (*P* < 0.0500, 7.89 ± 1.68, 6.54 ± 3.84, 1.22 ± 0.30 vs. 12.09 ± 5.47 respectively), while there was no statistically significant difference observed between USGroup and PosGroup (*P* = 0.6365, 11.02 ± 4.98 vs. 12.09 ± 5.47).

### Analysis for sCD36 in plasma

**Figure 3:**
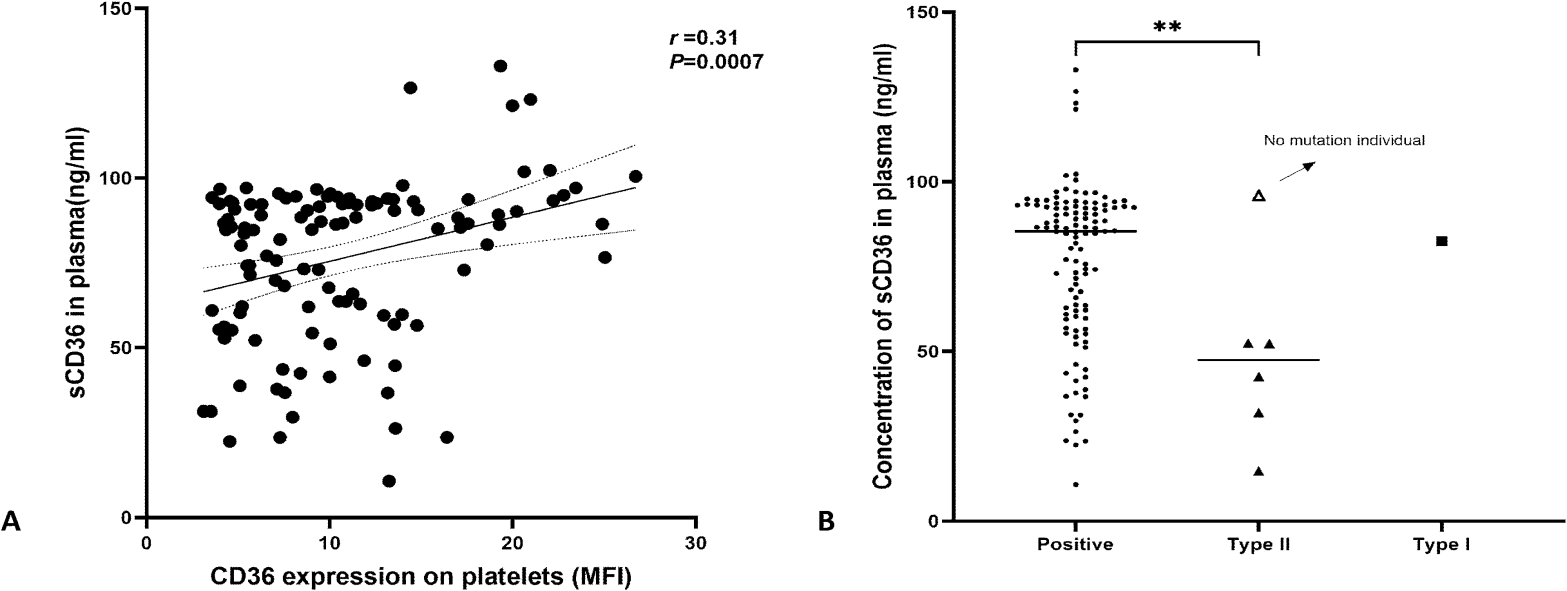
Analysis for sCD36 in plasma. (**A**) Correlation analysis between sCD36 in plasma and CD36 on platelet (N = 116). Abscissa and vertical axis represent the CD36 expression on the platelet by flow cytometry (MFI) and sCD36 level in plasma (ng/ml), respectively; (**B**) sCD36 comparison between CD36 positive and deficient individuals. The hollow triangle indicated by the arrow in the Type II group represents the individual with type II phenotype, but no mutation was detected on the sequencing region. “**”represent *P* < 0.0100.

We assembled a cohort of 122 plasma samples, which encompassed 7 CD36-deficient individuals (type I and type II) and 115 CD36-positive individuals. Our primary objective was to explore the correlation between CD36 expression on platelets and sCD36 in plasma. To accomplish this, we established a linear regression model employing the CD36-positive specimens. The results revealed statistically significant analysis, but indicated no correlation between sCD36 and CD36 expression on platelets (*r* = 0.31, *P* = 0.0007, N = 115) (Fig. 3A). Furthermore, we conducted a comparative analysis of sCD36 levels between CD36-positive and CD36-deficient individuals. It was evident that sCD36 levels among type II deficient individuals (N = 6) were notably lower than those of the positive controls (*P* = 0.0060, 48.36 ± 27.30 ng/ml vs. 76.39 ± 23.78 ng/ml) (Fig. 3B). Interestingly, one of the type II individuals was without mutations in the CD36 exons^16^, exhibited a higher sCD36 level in comparison to the 5 type II individuals harboring mutations [96.00 ng/ml vs. 38.83 ng/ml (mean value)] (Fig. 3B). In addition, sCD36 was also detected in the plasma of the type I individual (82.43 ng/ml).

### qPCR analysis

**Figure 4:**
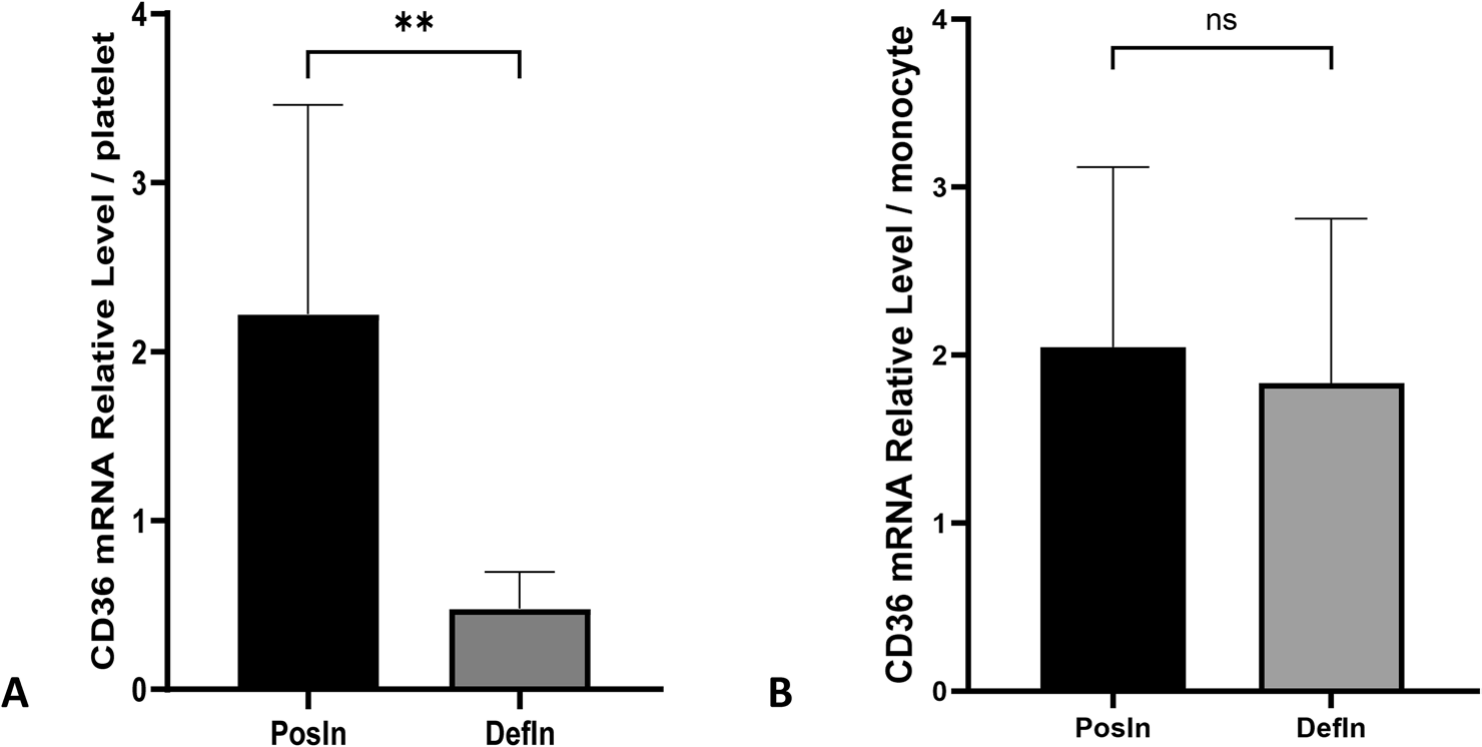
mRNA level analysis for platelet and monocyte. Posln and Defln respectively represent individuals with CD36 expression and CD36 type II deficiency. (**A**) Analysis for CD36 cDNA extracted from platelet. “**” indicates *P* = 0.0065; **(B**) analysis for CD36 cDNA extracted from monocyte. “ns” indicates *P* = 0.7500.

To analyze heterogeneity comprehensively, we employed the qPCR method to indirectly assess CD36 mRNA levels via cDNA detection. We selected a sample of 20 individuals, comprising 15 CD36-positive subjects (PosIn) and 5 individuals with type II CD36 deficiency (DefIn). Our initial focus was to compare CD36 mRNA levels derived from platelets among these three groups (Fig. 4A). The results demonstrated a significant difference, with CD36 mRNA in the DefIn group markedly lower in comparison to the PosIn group (*P* = 0.0065, 0.48 ± 0.22 vs. 2.22 ± 1.24). Additionally, we extended our analysis to compare CD36 mRNA levels in monocytes between the PosIn and DefIn groups, but no significant difference was observed (*P* = 0.7500, 2.05 ± 1.07 vs. 1.83 ± 0.98) (Fig. 4B).

## Discussion

The *CD36* gene comprises 15 exons, encompassing untranslated regions (exon1, 2, and 15) and protein-coding sequence (exon3 to 14). Most of mutations relevant to CD36 deficiency are location on protein-coding exons, while portions may be found on the alternative splicing regions^16^. Xu et al.^17^ found variations on 5ʹ UTR was supposed to lower the CD36 level, but there is no any report demonstrates the mutation on non-coding exons can cause CD36 deficiency. Therefore, in our study, we investigated protein-coding sequence polymorphisms in CD36 and identified 15 mutations. Significantly, one of these mutations, c.1329_1354*dup*GATAGAAATGATCTTACTCAGTGTTG, had not been previously documented in the dbSNP database. Noteworthy among the observed polymorphisms in CD36-positive subjects, only c.268C>T, c.410T>C, c.1156C>T, and c.1228_1239*del*ATTGTGCCTATT displayed an inclination towards the manifestation of CD36 deficiency^5,15,28,30^. Moreover, our investigation revealed that in comparison to earlier studies^17,31^, c.1228_1239*del*ATTGTGCCTATT is the most prevalent mutation. In contrast, mutations such as c.329_330*del*AC exhibited a notably lower allele frequency. Given our findings, particular attention should be devoted to the screening of c.1228_1239*del*ATTGTGCCTATT, especially within the context of CD36 deficiency in the Kunming platelet donor population.

Moreover, the available evidence concerning other SNP, particularly those situated in the ectodomain coding region remains limited. We conducted a systematic assessment of these SNPs using the HotMuSiC, CUPSAT, SAAFEC-SEQ and FoldX tools. These 4 analysis tools are databases used to predict the effects of mutations on protein stability by integrating empirical energy functions, statistical potentials, atomic force data, and structural information, prioritizing mutations for experimental validation and optimizing protein stability for various applications^24–27^. In particular, FoldX is notably distinguished for its superior predictive capabilities in estimating the effects of mutations on protein stability^27^. The results of this study revealed a notable reduction in the stability of CD36. Remarkably, this prediction aligns with our observations from flow cytometry, which consistently showed a decrease in MFI (Table 2; Fig. 2). While this bioinformatic prediction provides valuable insights into the potential association between SNPs and CD36 deficiency, it is imperative to conduct confirmatory experiments at the cellular level to validate these findings.

CD36 deficiency manifests in two subgroups, type I and type II. The underlying mechanistic basis of type II deficiency remains largely elusive. A widely recognized theory put forward by Kashiwagi^32^ proposes the existence of a "platelet-specific silent allele". However, this hypothesis falls short in explaining type II deficiency in individuals lacking any detectable mutations. Our previous study successfully amplified the truncated CD36 transcript derived from platelets and we inclined that the alteration in RNA level may give us more clues to understand type II phenotype^16^. In this study, we applied qPCR to analyzed CD36 mRNA levels in both platelets and monocytes. As expected, qPCR results conclusively demonstrated that CD36 mRNA persists in platelets of type II individuals, albeit at a diminished level. Meanwhile, we also detected the CD36 mRNA levels in the type I deficient individual (ΔΔCt value: 0.45). However, we only screened one donor, and the sample size is insufficient to conduct a statistical analysis. When we integrate these findings with our previous work involving a non-mutation type II individual, we propose an optimized explanation of the "platelet-specific silent allele" theory. One of the alleles remains silenced, while the other continues to transcribe CD36 mRNA but undergoes alternative splicing as well as decrease of RNA level, a process potentially governed by enigmatic regulatory mechanisms.

In our comparative analysis of protein levels using MFI, we observed a significant reduction in CD36 protein expression in individuals harboring CD36 deficiency-associated mutations, consistent with previous research^15,17^. Notably, flow cytometry serves as the predominant technique for CD36 deficiency detection, albeit with relatively demanding sample and equipment requirements. To address these challenges, Phuangtham et al. proposed the utility of sCD36 as a potential biomarker for CD36-deficient individuals. Their study revealed the absence of detectable sCD36 in the plasma of type I deficient individuals and demonstrated a strong correlation between sCD36 levels in plasma and CD36 expression on platelets (r^2^ = 0.8551)^23^. In our study, we also incorporated sCD36 detection into our research. Intriguingly, our study did not find such a good correlation, and different sample size may be the main reason for this disparity. However, Peter Wilhelmsen et al.^33^ also demonstrated that there may be no correlation between CD36 on platelets and sCD36 in plasma. Besides, we also detected sCD36 in one individual exhibiting a type I phenotype, a departure from Phuangtham's findings. This variance can be attributed to genotype differences. Specifically, the type I individual in our study was in a heterozygous state (c.268 C>T) but with no CD36 protein expression on platelet and monocyte caused by only mutant transcript occurrence^16^, while wild-type CD36 mRNA may present in other cell types and sCD36 could be secreted. Moreover, speculative evidence from Kashiwagi suggests that type I deficient individuals with c.268C>T homozygosity fail to produce CD36 antibodies when exposed to positive antigens, possibly due to the limited presence of antigens on the cell surface^28,34^. Consequently, we hypothesize that the development of CD36 antibodies may not occur after exposure to positive antigens in the type I individual identified in our donor cohort. Conversely, Phuangtham et al.^23^ found no significant difference in sCD36 levels between type II and CD36-positive individuals, whereas our results revealed a contrary significance. These discrepant outcomes can also be attributed to different genotypic profiles. In our studies, type II individuals without mutations exhibited higher sCD36 levels compared to those harboring mutations. In Phuangtham's study, however, 4 out of 6 type II individuals without mutation, while only one individual among type II phenotypes displayed no mutations in our study. Furthermore, the majority of reported CD36 antibody-mediated severe diseases have been associated with type I individuals, with limited literature addressing type II phenotypes. In conclusion, we posit that sCD36 detection may offer advantages in assessing the risk of immune reactions in CD36-deficient individuals. Nonetheless, additional clinical trials and fundamental research are warranted to substantiate these findings.

In summary, this study investigates CD36 gene mutations associated with CD36 deficiency. We found 15 mutations, including a novel one (c.1329_1354dupGATAGAAATGATCTTACTCAGTGTTG). Four mutations (c.268C>T, c.410T>C, c.1156C>T, and c.1228_1239delATTGTGCCTATT) are linked to CD36 deficiency. Especially, the prevalence of c.1228_1239delATTGTGCCTATT, the most frequent genetic variant associated with CD36 deficiency. Therefore, special attention should be dedicated to this variant during screenings for CD36 deficiency. Bioinformatics analysis confirms reduced CD36 stability for specific mutations. The study also proposes an optimized interpretation of the "platelet-specific silent allele" theory for type II CD36 deficiency. Additionally, it suggests sCD36 as a potential biomarker for immune reactions in CD36-deficient individuals but calls for further research to validate these findings.

## Data Availability

The datasets used and/or analysed during the current study available from the corresponding author on reasonable request.
